# Levofloxacin-ceftazidime administration regimens combat *Pseudomonas aeruginosa* in the hollow-fiber infection model simulating abnormal renal function in critically ill patients

**DOI:** 10.1186/s40360-020-0396-5

**Published:** 2020-03-04

**Authors:** L Zhao, X Li, X He, L Jian

**Affiliations:** Department of Pharmacy, Shengjing Hospital of China Medical University, Shenyang, 110004 China

**Keywords:** Levofloxacin, Ceftazidime, Hollow-fiber infection model, Renal function, *Pseudomonas aeruginosa*

## Abstract

**Background:**

The purpose of this study was to investigate the bactericidal effects of levofloxacin and ceftazidime as both monotherapy and combination therapy, and to determine their effects on resistance suppression in patients with normal and abnormal (Ccr:16–20 mL/min) renal function. Common clinical administration regimens to provide reference values were further evaluated.

**Methods:**

The 7-d hollow-fiber infection model was used to inject the *Pseudomonas aeruginosa* standard strain (ATCC27853), which simulated common clinical administration regimens for patients with different renal function. Ten regimens were stratified into 2 categories based on renal function, and each category contained 3 monotherapy regimens and 2 combination therapy regimens. Total and resistant populations were quantified. Drug concentrations were determined by high-performance liquid chromatography (HPLC).

**Results:**

Monotherapy regimens resulted in about 0.5-log-CFU/mL bacterial kill in the total population at 6 or 8 h, whilst combination regimens resulted in 2- to 3-log-CFU/mL within 2 days. For levofloxacin monotherapy regimens in patients with normal renal function, resistance emergence was seen after 6 h, and was seen at 0 h in the ceftazidime monotherapy regimen, as well as in all regimens of patients with abnormal renal function. Although resistant subpopulation in combination regimens with abnormal renal function began to increase at 0 h, there was a definite downward trend after 8 h, while resistant population in the normal renal function group increased after 16 h.

**Conclusions:**

Combination therapy had greater bactericidal efficacy and resistance inhibition compared with monotherapy. Studying combination regimens in randomized clinical trials is warranted.

## Background

*P. aeruginosa* is a common conditional pathogen of hospital acquired pneumonia, especially in patients in intensive care units (ICUs) who require respiratory support [1]. Abnormal renal function is common in ICUs patients. Doses of antibacterial therapy need to be adjusted for ICUs patients. This is explained by the kidney is the main organ for drug elimination, so without dose adjustment, the accumulation of drugs and their metabolites in plasma would increase the possibility of toxicity [2]. At present, the resistance to *P. aeruginosa* is increasing [3]. About 15% of *P. aeruginosa* strains are resistant to at least three-fifths of antimicrobial agents with an antipseudomonal spectrum (ceftazidime, fluoroquinolones, piperacillin/tazobactam, carbapenems and aminoglycosides), and 5% are resistant to all 5 according to data from the European Centre for Disease Prevention and Control [4]. Combined with clinical practice, the main cause of bacterial resistance is the unreasonable use or even abuse of antibacterial drugs. In the process of exploring how to reasonably use antibiotics and reduce bacterial resistance, the establishment of the in vitro hollow-fiber infection model (HFIM) has become an important research method.

Yadav et al. [5] used HFIM to optimize the combination of piperacillin and tobramycin, and their research showed that optimized combination regimens brought out effective bacterial kill and inhibition of resistance emergence in patients with augmented renal clearance. Jumbe et al. [6] demonstrated that levofloxacin can inhibit amplification of resistant subpopulations when the AUC/MIC (area under the concentration-time curve at 24 h divided by minimum inhibitory concentration) ratio reaches 157. However, in patients treated with 750 mg levofloxacin once daily, when Monte Carlo simulation was used to detect the frequency of achieving this ratio in the distribution of levofloxacin MIC values, only 61% were able to reach it. In a meta-analysis including 17 studies, Vardakas et al. [7] demonstrated that the combination of levofloxacin and β-lactam increased the bacterial kill rate for community acquired pneumonia.

Therefore, it is important to explore combination therapy for rapid bacterial kill and inhibition of amplification of resistance. We explored the clinically relevant 7-day administration period of levofloxacin and ceftazidime against the *P. aeruginosa* standard strains using the HFIM.

## Methods

### Microorganisms and antimicrobials

This study was conducted in accordance with the Declaration of Helsinki. *P. aeruginosa* ATCC27853 was provided by the Laboratory of Shengjing Hospital affiliated to China Medical University, Shenyang, China. The strain was stored at − 70 °C. Levofloxacin (Daiichi Sankyo, batch number: BS099N1) and ceftazidime (GlaxoSmithKline, batch number: 16120020) were obtained from the Shengjing Hospital Pharmacy department.

### Susceptibility studies

The MIC was defined as the minimum concentration of drug that led to invisible growth after incubation at 37 °C for 24 h. The MICs of levofloxacin and ceftazidime for *P. aeruginosa* ATCC27853 were resolved by using the agar dilution and broth microdilution methods described by the Clinical and Laboratory Standards Institute (CLSI) [8]. The MPC (mutation preventive concentration) was determined at 72 h by using of agar dilution methods. Different concentrations of MHA (Mueller Hinton agar) plates containing levofloxacin and ceftazidime separately were prepared. The drug concentrations were combined in a checkerboard method (*n* = 3). The fractional inhibitory concentration (FIC) value was determined by the minimum concentration of levofloxacin and ceftazidime without visible growth after 24 h of incubation at 37 °C.

### High-performance liquid chromatography (HPLC) methods

A validated HPLC method was used to determine concentrations of levofloxacin and ceftazidime in samples acquired from the central reservoir. The column used was an Agilent ZORBAX SB-C18 (4.6 mm × 150 mm, particle size 5 μm). For levofloxacin, the mobile phase included 0.05 mol/L K_2_HPO_4_ (containing 0.3% triethylamine, adjusted to PH ≈ 3 by phosphoric acid) and acetonitrile (volume ratio = 89:11). The UV detector was used at 295 nm, column temperature was 30 °C, flow rate was 1 mL/min, injection amount was 50 μL. For ceftazidime, the mobile phase included 0.05 mol/L K_2_HPO_4_ (adjust to PH ≈ 5.8 by triethylamine) and methanol (volume ratio = 80:20). The UV detector was used at 254 nm, column temperature was 30 °C, flow rate was 1 mL/min, injection amount was 50 μL.

### Hollow-fiber infection model

Blaser [9] described the HFIM as a pharmacodynamic system for bacteria, and Bilello [10] introduced its schematic and description. In the current study, levofloxacin and/or ceftazidime were instilled into the central reservoir every 8 h (ceftazidime) or every day (levofloxacin). The infusion time was 60 or 90 min for levofloxacin, and 30 min for ceftazidime. In experiments where the 2 drugs were used at the same time, the half-lives of levofloxacin (about 6.4 h) and ceftazidime (about 1.9 h) were quite different, which was solved by the approach of Blaser [11]. MHB (Mueller Hinton broth, no drug) was instilled into the central reservoir from the diluent reservoir to dilute the drug, simulating the elimination of the drug in vivo. Partial MHB-containing drugs were taken out from the central reservoir simultaneously to maintain an equal volume system. Bacteria were inoculated and limited into the outside of capillary of the hollow fiber, and exposed to the fluctuating drug concentration in the central reservoir through an internal cyclic pump in the system.

### Dosage regimens and pharmacokinetic(PK) studies

The inoculum was prepared by a medium-sized colony of *P. aeruginosa* growing in MHB in a 37 °C on a constant temperature shaker at 200 r/min overnight to logarithmic growth phase. The appropriate amount of bacterial solution was diluted to 1–2 × 10^7^ CFU/mL and inoculated into a HFIM. There were ten experimental groups and 1 control group. Initial experiments were conducted to simulate common regimens for patients with normal renal function. For levofloxacin monotherapy, doses of 500 and 750 mg daily were administered as a 60 and 90 min infusion. For ceftazidime monotherapy, a total dose of 3 g was given as 1 g every 8 h, administered as 30 min infusions. The combination regimens were as follows. The 500 and 750 mg levofloxacin doses were combined with 1 g of ceftazidime every 8 h, respectively. The regimens of subsequent experiments were based on renal creatinine clearance. We selected the critically ill patients with Ccr of 16–20 mL/min to simulate the regimens. Corresponding regimens were 125 and 187.5 mg levofloxacin daily as 60 and 90 min infusions, and 1 g ceftazidime daily as a 30 min infusions. The 125 and 187.5 mg doses of levofloxacin were combined with 1 g of ceftazidime daily, respectively. Concentrations of levofloxacin and ceftazidime obtained in the experiment were measured using validated HPLC methods. Samples were taken from the central reservoir at different time points and stored at − 80 °C, then the concentration-time curve was plotted with Graphpad prism 8, and data was analyzed with SPSS 24.0.

### Pharmacodynamic (PD) studies

Bacterial culture samples were taken from the hollow-fiber cartridge at different time points. Serially diluted samples were quantitatively cultured onto drug-free MHA plates to count the total number of bacteria. Some bacterial samples were quantitatively cultured onto MHA plates with either levofloxacin at 5 × and 10 × MIC or ceftazidime at 5 × and 10 × MIC, or a combination of the 2 drugs at 1 × and 2 × FIC for *P. aeruginosa* ATCC27853, to assess the impact of each regimen on the resistant populations. MHA plates were incubated at 37 °C for 24 h before the results were read. Time-sterilization curves were then drawn, compared with Graphpad prism 8 and the data were analyzed with SPSS24.0.

## Results

### Susceptibility study results

The MICs for *P. aeruginosa* ATCC27853 were 0.5 μg/mL for levofloxacin and 1.0 μg/mL for ceftazidime. The MPCs for *P. aeruginosa* ATCC27853 were 5.1 μg/mL for levofloxacin and 82 μg/mL for ceftazidime. The FIC value of levofloxacin combined with ceftazidime was measured to be 1.0 (concentrations of levofloxacin and ceftazidime were 0.25 μg/mL and 0.5 μg/mL, respectively).

### High-performance liquid chromatography (HPLC) studies

The retention time for levofloxacin was 6–8 min and ceftazidime was 8–10 min. The assay of levofloxacin was linear over a range of 1.5625–50 μg/mL (y = 59.97x-24.055, r^2^ = 0.9998) and the assay of ceftazidime was linear over a range of 10–320 μg/mL (y = 0.4659x + 2.9403, r^2^ = 0.9999). The inter-day coefficients of variation for the quality control samples analyzed in triplicate at 3 concentrations on each analysis day ranged from 0.86–2.36% for levofloxacin and 0.67–1.61% for ceftazidime. The intra-day coefficients of variation ranged from 0.94–3.75% for levofloxacin and 0.33–1.46% for ceftazidime. Accuracies of levofloxacin and ceftazidime were 99.17–102.88% and 98.97–101.58%, respectively.

### Resistant mutation frequency

For *P. aeruginosa* ATCC27853, the average density of resistant subpopulation at 5 × and 10 × MIC of levofloxacin value was – 4.35 and – 3.48 log CFU, respectively. The average density of resistant subpopulation at 5 × and 10 × MIC of ceftazidime was – 5.01 and – 4.42 log CFU, respectively. At 1 × and 2 × FIC value, the average densities of resistant subpopulation were − 4.21 and – 2.96 log CFU, respectively.

### Pharmacokinetic (PK) results

**Fig. 1:**
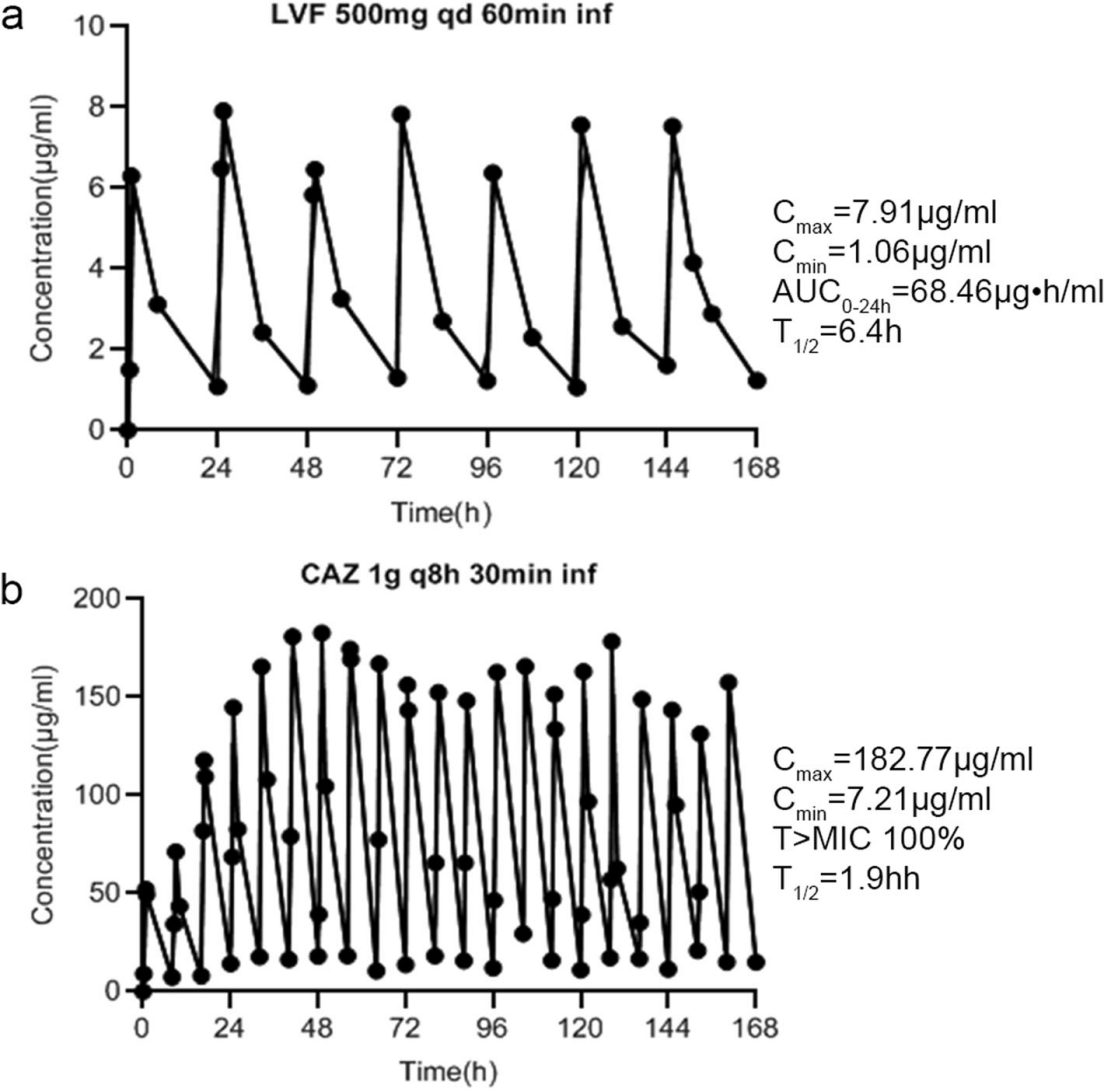
Various PK simulations in the study. T_1/2_, elimination half-life; AUC_0-24h_, area under the concentration-curve from time 0 to 24 h; C_max_, maximum drug concentration; C_min_, minimum drug concentration; T > MIC is the percentage of administration interval. **a** concentration-time curve of LVF 500mg qd 60min inf; **b**: concentration-time curve of CAZ 1g q8h 30min inf

Typical concentration-time curves for levofloxacin and ceftazidime are shown in Fig. 1a and b. Data for all regimens are available on request.

### Pharmacodynamic (PD) studies of monotherapy regimens

**Fig. 2:**
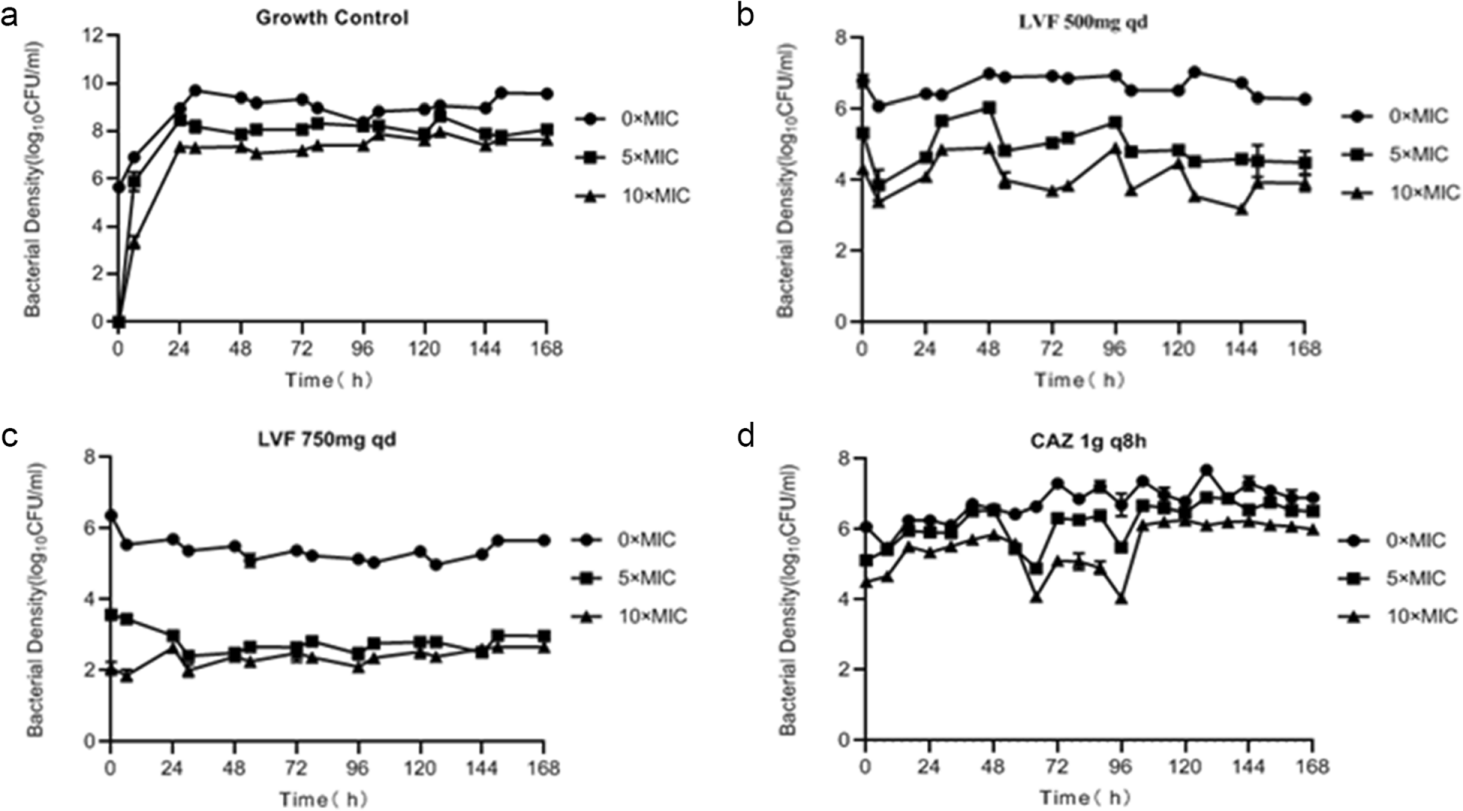
Observed time-sterilization curve of monotherapy regimens for patients with normal renal function. Data is expressed as the means ± SD of bacterial burdens. **a** time-sterilization curve of growth control; **b**: time-sterilization curve of LVF 500mg qd; **c**: time-sterilization curve of LVF 750mg qd; **d**: time-sterilization curve of CAZ 1g q8h

**Fig. 3:**
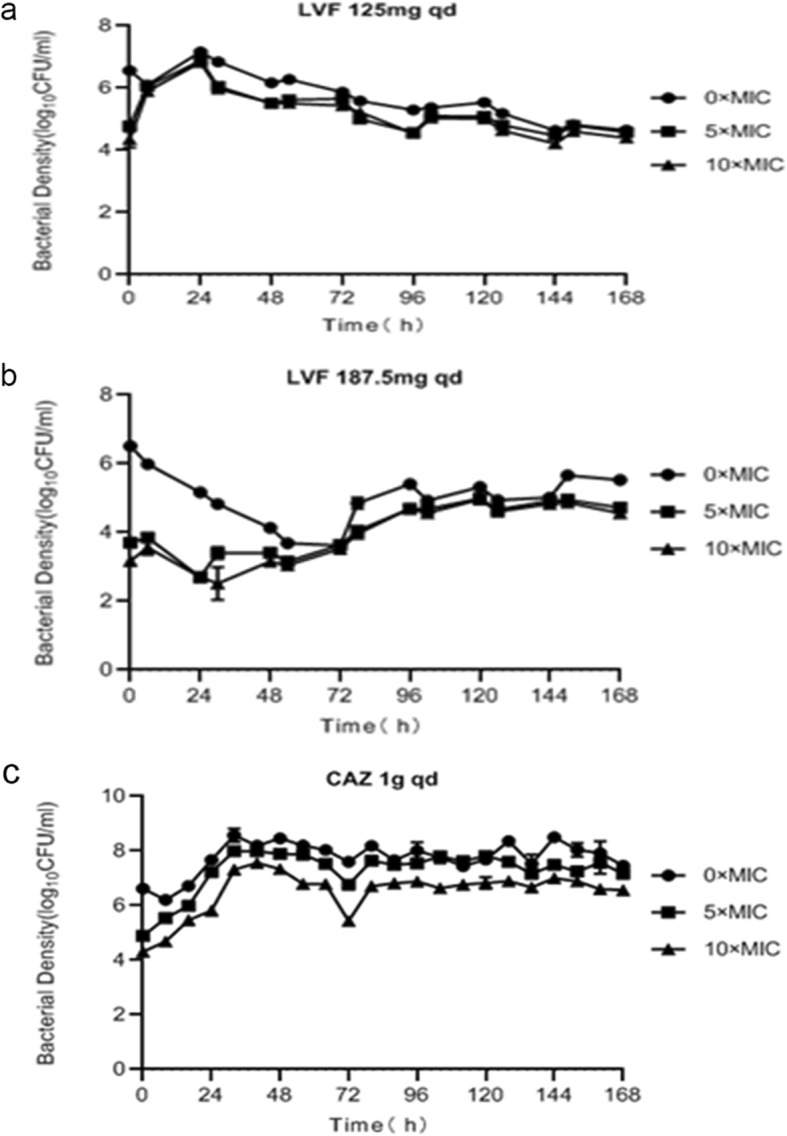
Observed time-sterilization curve of monotherapy regimens for patients with abnormal renal function. Data is expressed as the means ± SD of bacterial burdens. **a** time-sterilization curve of LVF 125mg qd; **b**: time-sterilization curve of LVF 187.5mg qd; **c**: time-sterilization curve of CAZ 1g qd

The monotherapy effects on total and resistant population burdens within 7 days are shown in Fig. 2 (control and normal renal function) and Fig. 3 (abnormal renal function). The bacteria grew well in the untreated growth control arms and reached a bacterial density of 10^9^ to 10^10^ CFU/mL by Day 2 (Fig. 2a). The effects of the levofloxacin and ceftazidime monotherapies for patients with normal renal function on the total and resistant population burdens for *P. aeruginosa* ATCC27853 are shown in Fig. 2b to d. The levofloxacin and ceftazidime control arms produced around a 0.5-log-CFU/mL reduction in the total population before 6 or 8 h; however, resistance was seen after 6 h for levofloxacin regimens and after 0 h for ceftazidime. Figure 3 displays the total and resistant population burdens for the levofloxacin and ceftazidime monotherapies for patients with abnormal renal function. The levofloxacin and ceftazidime control arms produced less than a 0.5-log-CFU/mL reduction in the total population before 6 or 8 h, and 187.5 mg levofloxacin showed a trend of reducing the total population until 72 h. However, rapid resistance emergence at 0 h was seen in the 3 regimens. Regeneration in the total number of the 6 monotherapy regimens shown in Fig. 2 and Fig. 3 can be explained by the presence of resistance.

### Pharmacodynamic (PD) studies of combination therapy regimens

**Fig. 4:**
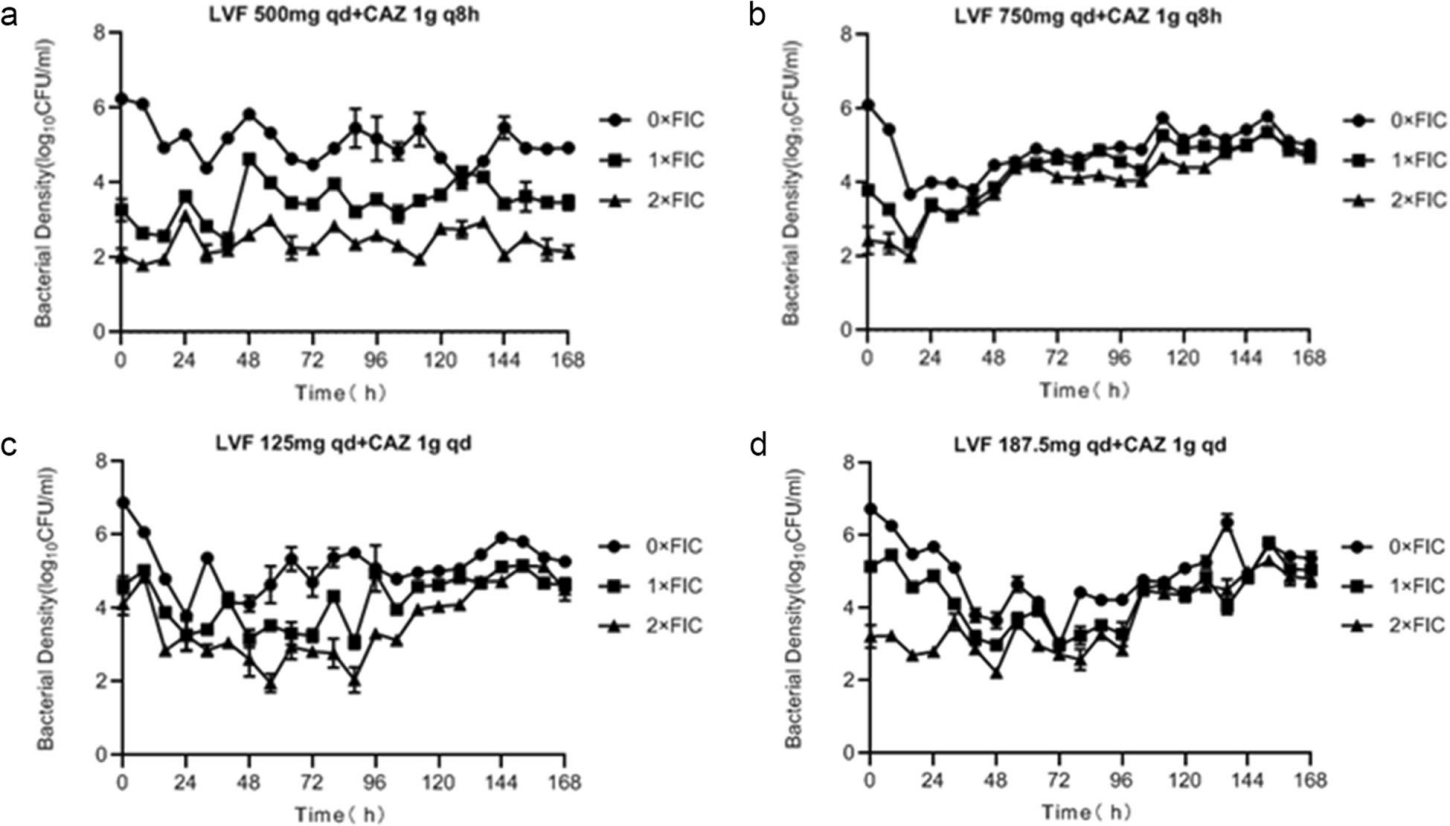
Observed time-sterilization curve of combination therapy regimens. Data is expressed as the means ± SD of bacterial burdens. **a** time-sterilization curve of LVF 500mg qd combined with CAZ 1g q8h; **b**: time-sterilization curve of LVF 750mg qd combined with CAZ 1g q8h; **c**: time-sterilization curve of LVF 125mg qd combined with CAZ 1g qd; **d**: time-sterilization curve of LVF 187.5mg qd combined with CAZ 1g qd

Figure 4 displays the efficacy of combination therapy on *P. aeruginosa* ATCC27853. There was a major effect of combination therapy, which resulted in a 2- to 3-log-CFU/mL bacterial kill on the total population. The growth of resistant bacteria was seen after 16 h in the patients with normal renal function, and at 0 h in the patients with abnormal renal function. But there was a downward trend after 8 h in the patients with abnormal renal function. Combination therapy regimens were able to suppress the resistant population amplification.

### Comparison of all regimens on total population burdens

**Fig. 5:**
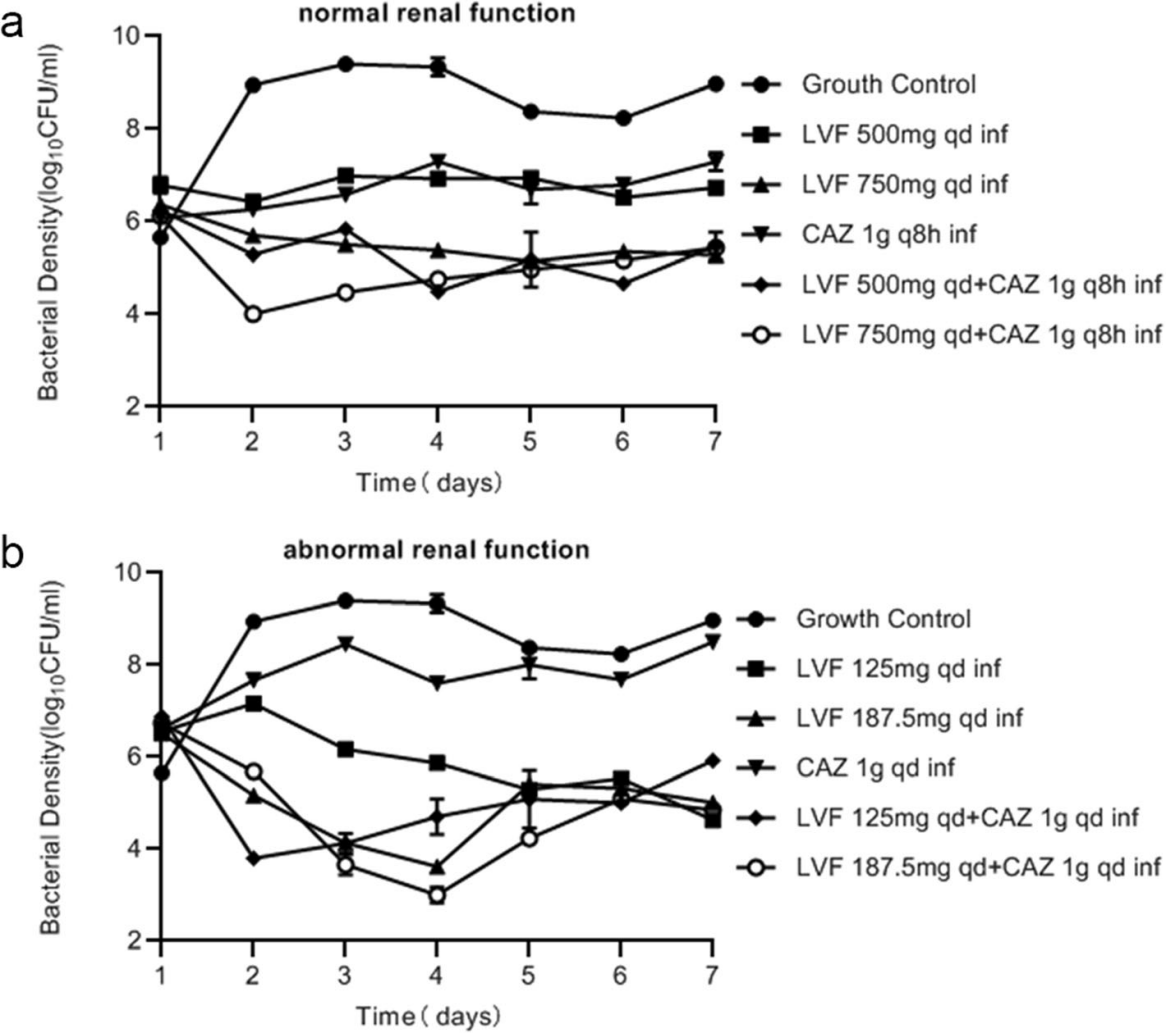
Comparison of total population burdens on all regimens. Data is expressed as the means ± SD of bacterial burdens. **a** comparison of total population burdens in patients with normal renal function; **b**: comparison of total population burdens in patients with abnormal renal function

Figure 5a shows the comparison of total population burdens in patients with normal renal function, and Fig. 5b shows the same for patients with abnormal renal function. Combination therapy resulted in a 2-log-CFU/mL bacterial kill in patients with normal renal function, and a 3-log-CFU/mL bacterial kill in patients with abnormal renal function. The results were clearly different from those seen with monotherapy (*p* < 0.01, analysis of variance). The comparison also showed that combination therapy was superior to monotherapy in resistance inhibition, because the regrowth in the total population for all regimens was expounded by resistant emergence.

## Discussion

The resistance rate of gram-negative bacteria is increasing. More importantly, it is reported that the multidrug resistance rate is alarmingly high, and steadily increasing [12, 13]. However, this is not surprising, as our treatment goals previously focused on maximizing clinical and microbiological cure rather than minimizing the emergence of antibiotic resistance [14]. According to the mutation selection window (MSW) theory proposed by Zhao [15], when the drug concentration is higher than the MIC value, antibacterial drugs can play an antibacterial role; however, when the concentration is between MIC and MPC value, while drugs play an antibacterial role, but they can also selectively enrich resistant mutants. Finally, when the concentration is higher than MPC value, resistant mutants must have ≥2 mutations at the same time for selective enrichment to occur. Therefore, we should not aim to treat patients based on clinical efficacy, but should also consider achieving inhibition of drug resistance on the basis of Zhao’s theory.

At present, the situation of bacterial resistance is severe. Luan’s study [16] showed that approximately 30% of *P. aeruginosa* was resistant when older outpatients were infected with community-acquired pneumonia. In a study by Fink et al. [17], monotherapy with 400 mg of ciprofloxacin administered every 8 h resulted in 33% resistance to hospital acquired pseudomonas pneumonia, whilst 500 mg of imipenem given every 6 h or 1000 mg every 8 h resulted in 50% resistance. The resistance rate of levofloxacin was 25% according to a study conducted by Shao Y [18]. In this context, we hope to explore the difference between monotherapy and combination therapy for *P. aeruginosa*.

The traditional combination idea is not to use drug combinations with shared mechanisms of resistance because the orthogonality of resistance probabilities will not hold. The emergence of β–lactamase (mainly including AmpC, ESBLs, MBLs, etc.) is the main mechanism of *P. aeruginosa*’s resistance to β–lactams [19]. Fluoroquinolones inhibit DNA gyrase (subunit composition: gyrA and gyrB) and topoisomerase IV (subunit composition: parC and parE) of bacteria [20], but the mutation of any these subunits leads to the resistance of bacteria to fluoroquinolones. As such, we chose to explore the combination of levofloxacin and ceftazidime. Although both drugs are excreted through the kidney, they are very common combination in the clinical treatment of patients with abnormal renal function combined with abnormal liver function and multidrug resistance patients. Up to our knowledge, this study is the first to use a HFIM to investigate the effects of the two drugs on bacterial kill and inhibition of resistance emergence in patients with abnormal renal function. We simulated monotherapy and combination therapy commonly used in clinic, and determined the administration regimens of patients with abnormal renal function according to the instructions. Although there may be some differences in the physiology, sensitivity of bacteria to drugs and binding efficiency of active proteins in HFIM or in vivo and animal studies, our study may better simulate patients with low immune function or without immune system (majority in ICUs) compared with normal patients, and make appropriate references to clinical rational use of drugs.

The PK of levofloxacin or ceftazidime was similar at the same dose in different groups of monotherapy and combination. When AUC_0-24h_/MIC≥100 and/or C_max_/MIC> 8, the bactericidal effect was good for fluoroquinolones, and when T > MIC is more than 50%, it showed a good bactericidal effect for β–lactams. However, as observed in some studies [21–23], treatment failure and rapid resistance emergence happened even when T > MIC reached or approached 100% for meropenem, or AUC_0-24h_/MIC was 168 for tobramycin which was 4 times of the suggested breakpoint. So it is necessary in order to find effective ways to inhibit drug resistance. The combination therapy had a better effect on the total and resistant population compared with monotherapy (Fig. 5). The monotherapy produced about a 0.5-log-CFU/mL reduction on the total population before 6 or 8 h (Figs. 2 and 3), while the combination therapy for patients with normal renal function achieved a 2-log-CFU/mL bacterial kill on total population, and reduced 3-log-CFU/ml for patients with abnormal renal function within 1–2 days (Fig. 4). Therefore, we can think of this combination as leading to synergistic sterilization. In addition, the effect of bacterial kill on combination therapy in patients with abnormal renal function was superior to that in patients with normal renal function. Because the combination of the two drugs has a potential cross effect on patients’ renal burden, this study may be useful for exploring lower doses of levofloxacin combined with ceftazidime to kill bacterial and inhibit resistance in patients with abnormal renal function.

For monotherapy, drug concentration exceeded the MPC value after the first dose when given as 500 or 750 mg levofloxacin once daily, which decreased to less than MPC after 6 h. As such, resistance emergence was seen after 6 h. Drug concentrations of other monotherapy regimens was between MIC and MPC after the first administration, so drugs were able to produce bactericidal effects, but not inhibit resistance, so resistance appeared at 0 h. For combination therapy, the growth of resistant bacteria appeared after 16 h in patients with normal renal function. In the patients with abnormal renal function, resistance emergence was seen at 0 h, but there was a downward trend after 8 h. Therefore, resistance may be reduced if the dose was increased properly to reach the MPC value at the first administration in patients with normal renal function. For patients with abnormal renal function, if their first dose was equal to that of normal patients, they may obtain better effects of bacterial kill and resistance suppression. Of course, the appropriate administration should be implemented according to patient’s Ccr later, and therapeutic drug monitoring should take place. In addition, we suggest priority should be given to combination rather than monotherapy.

## Conclusions

The combination of levofloxacin and ceftazidime is attractive, and the results of this study suggest their combined effects should be studied in randomized clinical trials to explore the lower doses for sterilization and resistance suppression.

## Supplementary information

**Additional file 1.**

## Data Availability

The datasets used and/or analysed during the current study are available from the corresponding author on reasonable request.

## Abbreviations

AUC: Area under the concentration-time curve
CAZ: ceftazidime
CLSI: Clinical and Laboratory Standards Institute
FIC: Fractional inhibitory concentration
HFIM: Hollow-fiber infection model
HPLC: High-performance liquid chromatography
ICUs: Intensive care units
LVF: Levofloxacin
MHA: Mueller Hinton agar
MHB: Mueller Hinton broth
MIC: Minimum inhibitory concentration
MPC: Mutation preventive concentration
MSW: Mutation selection window
PD: Pharmacodynamic
PK: Pharmacokinetic
Q8h: Quaque 8 h
Qd: Quaque day
