# Supplementary material for: Levofloxacin-ceftazidime administration regimens combat Pseudomonas aeruginosa in the hollow-fiber infection model simulating abnormal renal function in critically ill patients
Source: BMC Pharmacol Toxicol. 2020 Mar 4;21:20. doi: 10.1186/s40360-020-0396-5 (PMC7057547; doi:10.1186/s40360-020-0396-5)
Supplement: Supplementary file 1 — Additional file 1. [file 40360_2020_396_MOESM1_ESM.docx]

**Supplementary File**


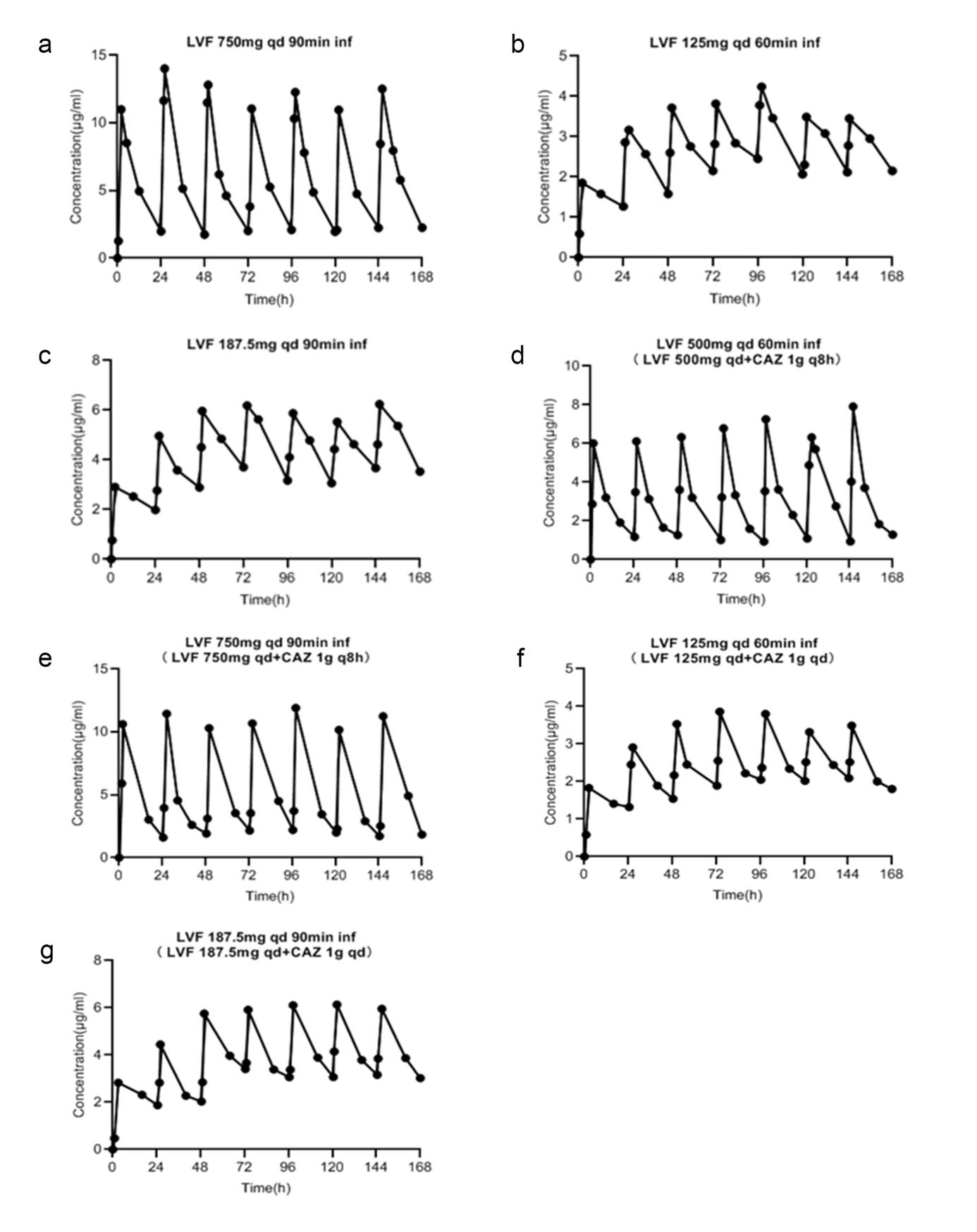


**Fig. 5** levofloxacin PK simulations in the study.


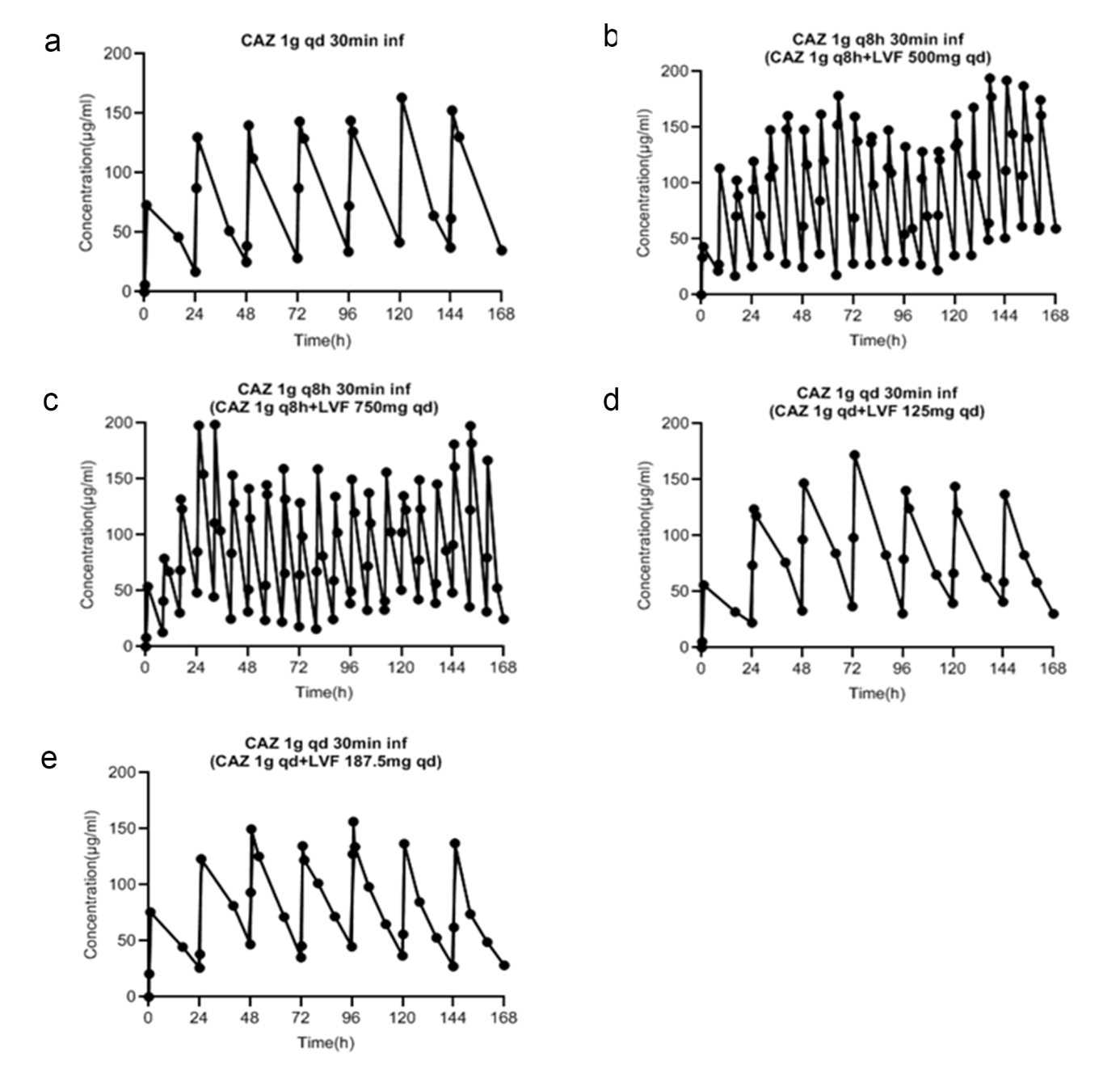


**Fig. 6** ceftazidime PK simulations in the study.
